# Mapping the conformational space accessible to catechol-*O*-methyltransferase

**DOI:** 10.1107/S1399004714012917

**Published:** 2014-07-25

**Authors:** Andreas Ehler, Jörg Benz, Daniel Schlatter, Markus G. Rudolph

**Affiliations:** a Molecular Design and Chemical Biology, F. Hoffmann-La Roche, Grenzacher Strasse 124, Basel, Switzerland

**Keywords:** catechol-*O*-methyltransferase, neurotransmitter degradation, catecholamine metabolism, SAM, *S*-adenosylmethionine

## Abstract

Methylation catalysed by catechol-*O*-methyltransferase (COMT) is the main pathway of catechol neurotransmitter deactivation in the prefrontal cortex. Low levels of this class of neurotransmitters are held to be causative of diseases such as schizophrenia, depression and Parkinson’s disease. Inhibition of COMT may increase neurotransmitter levels, thus offering a route for treatment. Structure-based drug design hitherto seems to be based on the closed enzyme conformation. Here, a set of apo, semi-holo, holo and Michaelis form crystal structures are described that define the conformational space available to COMT and that include likely intermediates along the catalytic pathway. Domain swaps and sizeable loop movements around the active site testify to the flexibility of this enzyme, rendering COMT a difficult drug target. The low affinity of the co-substrate *S*-adenosylmethionine and the large conformational changes involved during catalysis highlight significant energetic investment to achieve the closed conformation. Since each conformation of COMT is a *bona fide* target for inhibitors, other states than the closed conformation may be promising to address. Crystallographic data for an alternative avenue of COMT inhibition, *i.e.* locking of the apo state by an inhibitor, are presented. The set of COMT structures may prove to be useful for the development of novel classes of inhibitors.

## Abstract 2

Crystal structures of mouse, rat and human catechol-*O*-methyltransferase were determined in apo, semi-holo, holo and Michaelis forms under a variety of conditions. Domain swaps and large conformational changes in the active sites are observed, which testify to why this enzyme is a difficult drug target.

## 1. Introduction^1^   

An imbalance, usually low levels, of catechol-type neurotransmitters (NT) such as norepinephrine and dopamine is associated with severe conditions including Parkinson’s disease, depression and schizophrenia. In the brain, catechol-type NT are removed from the synapse by three main routes. While in the striatum these NT are predominantly taken up into the neuron by the dopamine transporter, enzymes are predominantly responsible for their inactivation in the prefrontal cortex by oxidation (monoamine oxidase and aldehyde dehydrogenase) and/or methylation (catechol-*O*-methyltransferase; COMT; Männistö & Kaakkola, 1999 ▶). Since inhibition of COMT enhances dopamine levels in the brain (Männistö & Kaakkola, 1999 ▶; Männistö *et al.*, 1992 ▶; McCarthy, 2001 ▶), an elevation of dopamine levels in the prefrontal cortex may prove effective in alleviating the symptoms of schizophrenia (Gupta *et al.*, 2011 ▶).

**Figure 1:**
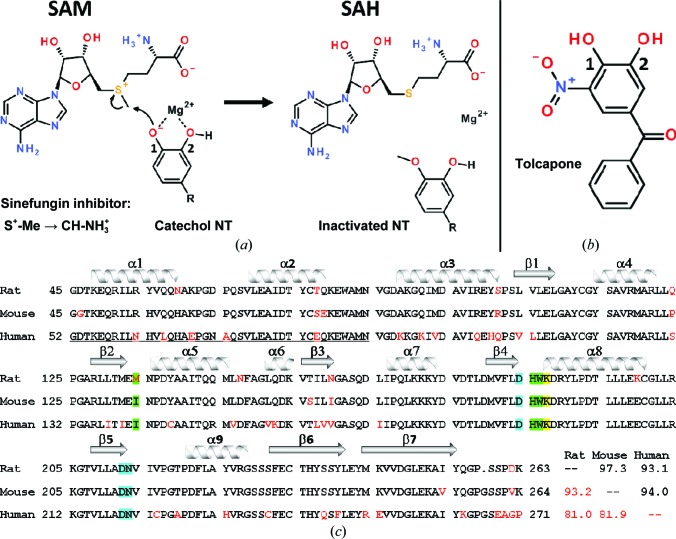
Sequence–catalysis relationship of COMT. (*a*) Scheme of the catalysed reaction. A catechol NT is bound as a bidentate ligand to an Mg^2+^ ion and deprotonated to form a nucleophile that attacks the activated methyl group of SAM. The methylated NT is not able to complex Mg^2+^ and dissociates. The natural product and antibiotic sinefungin is an isoster of SAM, thus acting as an inhibitor of many SAM-dependent enzymes [*e.g.* structures (7) and (8)]. (*b*) Structure of tolcapone, an acidic catechol and COMT inhibitor. The modified and unmodified hydroxyl groups of the catechol are indicated by the numbers 1 and 2, respectively. (*c*) Sequence alignment of the catalytic domains of rat, mouse and human COMT. The N-­terminal transmembrane sequences were ignored for the alignment. Sequence numbering for human COMT is shifted by seven with respect to the rodent enzymes. Secondary-structure elements are drawn at the top of the alignment. At the end of the alignment, red and black numbers are the percentage identity and similarity, respectively, between the sequence pairings. Red letters denote differences in at least one of the three sequences. Residues in the adenine and Mg^2+^-binding sites are highlighted with green and cyan backgrounds, respectively. The catalytic lysine residue has a yellow background.

COMT is a single-domain Mg^2+^-dependent methyltransferase that catalyses the transfer of an activated methyl group from *S*-adenosylmethionine (SAM) to its catechol substrate (Vidgren *et al.*, 1994 ▶; Guldberg & Marsden, 1975 ▶). The catechol NT is thus inactivated and SAM is converted to *S*-adenosylhomocysteine (SAH; Fig. 1 ▶ *a*). This reaction can be blocked by acidic catechols such as tolcapone that contain a nitro group (Fig. 1 ▶ *b*). The crystal structure of COMT comprises a central β-sheet flanked by α-helices, constructing a bipartite active site that accommodates the methyl donor SAM (in the so-called adenine site) and that can also complex an Mg^2+^ ion to which the catechol substrates bind (Vidgren *et al.*, 1994 ▶). An array of crystal structures of human and rat COMT have been determined, all of them in complex with substrates, products or inhibitors (Ellermann *et al.*, 2009 ▶, 2011 ▶, 2012 ▶; Tsuji *et al.*, 2009 ▶; Rutherford *et al.*, 2008 ▶; Palma *et al.*, 2006 ▶; Bonifácio *et al.*, 2002 ▶; Lerner *et al.*, 2001 ▶; Vidgren *et al.*, 1994 ▶). The first insight into the mechanism of methyl transfer was obtained from the co-crystal structure of rat COMT in complex with SAM and an unreactive dinitrocatechol. This surrogate of the Michaelis complex showed the precise pre-organization of one of the Mg^2+^-bound catechol hydroxyl groups with respect to the electrophilic methyl group of SAM (Vidgren *et al.*, 1994 ▶). This close arrangement also spawned the design and structural characterization of high-affinity bisubstrate inhibitors that span both halves of the active site (Ellermann *et al.*, 2009 ▶, 2011 ▶, 2012 ▶; Paulini *et al.*, 2004 ▶, 2006 ▶; Lerner *et al.*, 2001 ▶, 2003 ▶; Masjost *et al.*, 2000 ▶).

With a *k*_cat_ value of 24 min^−1^ for the rat liver isoform, COMT is a rather slow enzyme (Vidgren *et al.*, 1994 ▶). Based on the large volume and flexibility of SAM, which is entirely buried within COMT, and the necessity of exchanging the product SAH for SAM to enable further catalytic cycles, sizeable conformational changes in COMT that might explain the slow turnover were predicted early on (Vidgren *et al.*, 1994 ▶). An ordered bi-bi mechanism with SAM binding first to COMT followed by the catechol substrate was reported (Tunnicliff & Ngo, 1983 ▶) and indicates that the apo form, the semi-holo form with SAM bound (but not Mg^2+^) and the holo form with SAM and Mg^2+^ bound are all obligatory intermediates along the reaction coordinate.

Unfortunately, structures were lacking of COMT in the apo, semi-holo or holo forms which, together with the known structure of the Michaelis complex, could draw a picture of the conformational changes that possibly occur during catalysis. We have determined such structures for rat COMT in a variety of conditions and crystal forms. Sizeable conformational differences are observed that are not dominated by crystal contacts and that are relevant to catalysis. In addition, several domain-swapped dimers of human COMT were obtained and are compared with domain-swapped dimers of rat COMT. These results establish that COMT is quite a malleable enzyme, which renders it a difficult drug target. Binding energy from COMT–ligand interactions is lost by fixing certain conformations, which is entropically disfavoured. We also discuss the first structure of mouse COMT (>80% sequence identity; Fig. 1 ▶ *c*), which confirms a conserved core that is decorated with mobile loop regions, similar to the rat and human homologues. The observed conformational differences of loops near the mouse, rat and human COMT catalytic centres provide insight into catalysis and indicate a possible novel strategy for inhibitor development.

## 2. Materials and methods   

### 2.1. Purification, crystallization, data collection, phasing and refinement   

The cloning, production and purification of soluble N-terminally truncated mouse, rat and human COMT was performed as described previously for the rat and human isoforms (see the Supplementary Information of Ellermann *et al.*, 2009 ▶). The soluble form of COMT lacks the N-terminal 43 residues that constitute a single-pass helical membrane anchor. Residue numbering follows that of full-length COMT. The active sites of human and rat COMT differ at rat positions 134 and 138 (91 and 95 in rat numbering for the soluble form), where human COMT has Ile and Cys and rat COMT has Met and Tyr, respectively. Since rat COMT crystallizes more readily than human COMT, humanized rat COMT (hrCOMT, M134I/Y138C) was used as a surrogate for the human active site.

A detailed list of all crystallization conditions is given in Supplementary Table S1^2^. Crystals, data sets and coordinates are addressed by the numbers (1)–(12) throughout the text. Prior to data collection, crystals containing less than 20% PEG of any kind were cryoprotected using a quick dip in paraffin oil. All crystals were flash-cooled in liquid N_2_ by hyperquenching (Warkentin & Thorne, 2007 ▶). Data with high-resolution limits between 1.2 and 2.6 Å [based on *I*/σ(*I*) ≥ 1.0 and CC_1/2_ > 0.2; Supplementary Table S1] were collected at 100 K on beamline X10SA at the Swiss Light Source with PILATUS 6M pixel or MAR225 CCD detectors using X-rays of 1 Å wavelength. Data were integrated with *XDS* (Kabsch, 2010 ▶) and scaled with *XSCALE* (Kabsch, 2010 ▶) for (9), *SCALA* (Evans, 2006 ▶) for (7) or *SADABS* (Bruker) for all others. All structures were determined by molecular replacement in *Phaser* (McCoy *et al.*, 2007 ▶) using the highest resulting LLG from an array of in-house search models. Models were rebuilt in *Coot* (Emsley *et al.*, 2010 ▶) and refined with *PHENIX* (Zwart *et al.*, 2008 ▶) or *BUSTER* in the case of (5). The diffraction data for (5) in space group *P*3_2_21 exhibited signs of hemihedral twinning as judged by a decreased |*E* ^2^ − 1| value of 0.572 (expected value of 0.736) and a slightly sigmoidal shape of the cumulative intensity distribution (in the absence of pseudo-translation). Despite the low standard deviation for the normalized structure-factor amplitudes, twinning could be ignored during refinement (estimated twin fraction of 0.12 from a Britton plot with operator −*h*, −*k*, *l*). The diffraction data for (12) in space group *R*3:*H* are perfectly merohedrally twinned (|*E* ^2^ − 1| = 0.566, operator *k*, *h*, −*l*) and emulate *R*32:*H*. Twinning was taken into account during refinement. The final model contains two NCS-related protomers in the asymmetric unit. The NCS axis is perpendicular to the crystallographic threefold but does not intersect with it, excluding the higher symmetry *R*32:*H* setting. All refinement statistics are collected in Supplementary Table S2 and representative OMIT maps for selected ligands are shown in Supplementary Fig. S1. Coordinates and structure factors have been deposited in the Protein Data Bank (see the tables for PDB codes).

## 3. Results and discussion   

With a single exception (PDB entry 2zlb; Tsuji *et al.*, 2009 ▶), all of the currently known crystal structures of COMT have been determined in complex with substrates (SAM), products (SAH), inhibitors (sinefungin and nitrocatechols) or bisubstrate inhibitors. The latter span from the so-called adenine site to the Mg^2+^ site, which bind the substrates SAM and catechol, respectively (Lerner *et al.*, 2001 ▶; Ellermann *et al.*, 2009 ▶, 2011 ▶, 2012 ▶). The conformational variations within the inhibitor-bound structures are limited as they mostly represent a closed conformation in which both substrate-binding sites are occupied. The first insight into the conformational plasticity of COMT was provided by the apo structure of rat COMT (Tsuji *et al.*, 2009 ▶), which showed altered α2/α3 and β6/β7 loop regions compared with a SAM-bound form. Here, we extend this view by determination of the apo structures of human, rat and mouse COMT (summarized in Table 1 ▶).

### 3.1. Apo structures of human and mouse COMT identify three mobile loops   

**Figure 2:**
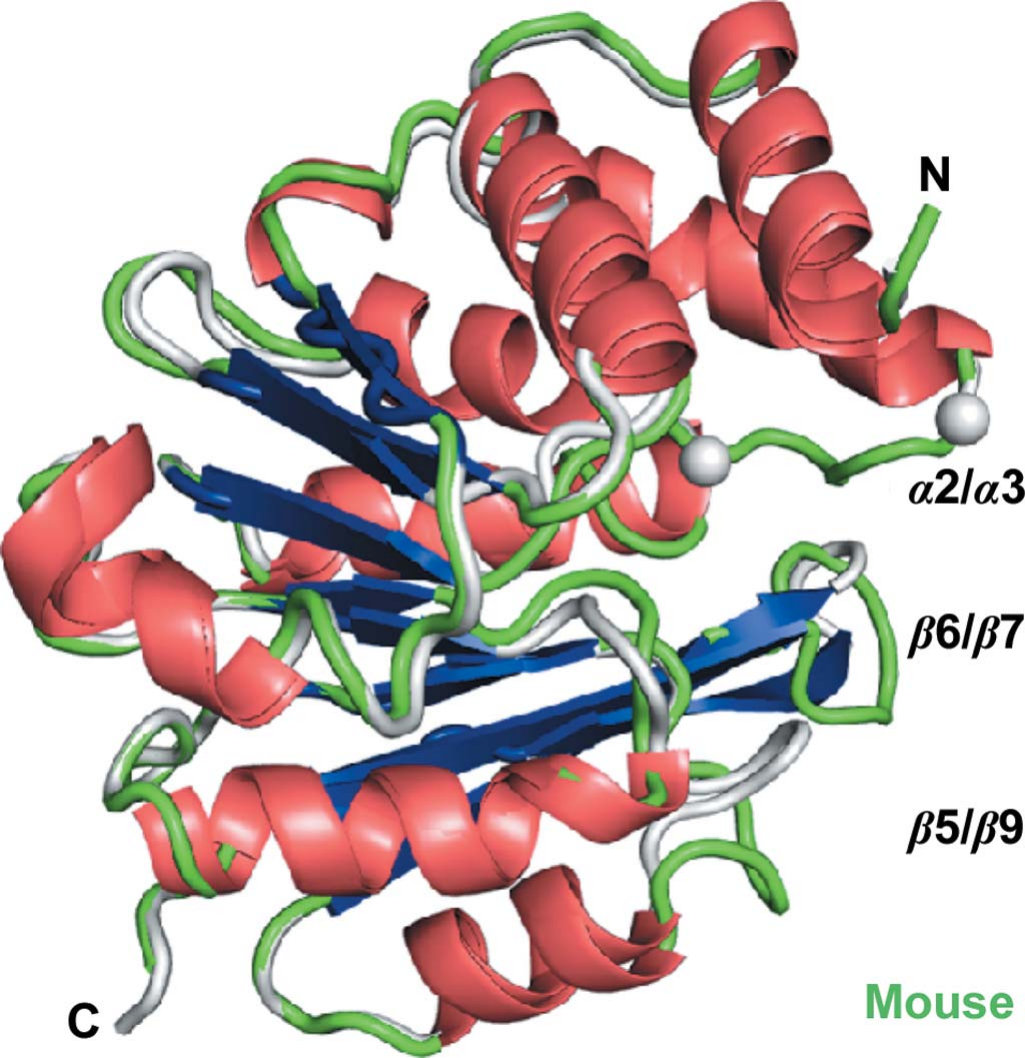
Novel human and mouse apo COMT crystal structures. The ribbon diagrams of human apo COMT (1) and mouse apo COMT (4) show a central seven-stranded β-sheet that is flanked by α-helices. The termini are marked, and three loop regions (grey for human COMT and green for mouse COMT) that are important for catalysis and for domain swapping are labelled α2/α3, β5/α9 and β6/β7. α2/α3 is disordered in this apo form of human COMT (indicated by grey spheres) but is ordered in mouse COMT.

A potential problem for *in vivo* data gained from mouse models that are not transgenic for human COMT is differences in the structures or activities of the homologues. In order to assure that the binding mode of compounds to mouse COMT is the same as that for rat and human COMT, it was hoped to determine a number of mouse COMT crystal structures in complex with selected inhibitors. However, in our hands mouse COMT proved to be difficult to crystallize and only an apo structure (4) was obtained. As expected by the high sequence similarity of >93% between the three orthologues (Fig. 1 ▶ *c*), the mouse COMT structure (4) reiterates all of the salient features of the rat and human COMT structures. However, when comparing this apo form with a human COMT apo structure (1), three loop regions that adopt different conformations in human and mouse COMT are identified (Fig. 2 ▶). In human COMT (1) the α2/α3 loop is disordered, but it is well defined in mouse COMT. Loop regions β5/α9 and β6/β7 are well defined but differ by distances of up to 8 Å at their tips (Fig. 2 ▶). Importantly, in the mouse COMT structure (4) these three loop regions adopt the same conformations as in the rat COMT structures (5) and (6) (see below). While it is possible that the apo state of COMT can adopt several conformations owing to an absence of conformational restrictions on the active-site loops, the recurrence of the same state in rat and mouse COMT points to a stable state of this apo form that is independent of crystal form and organism and that therefore may be adopted by COMT during catalysis after the reaction products have dissociated.

### 3.2. Domain swapping in human and rat COMT suggests high plasticity   

The structural plasticity of COMT is not limited to loops α2/α3, β5/α9 and β6/β7: entire sub-domains can be swapped to form COMT dimers. At present, it is unclear, if not doubtful, whether domain swapping has a biological relevance, but the presence of such swaps indicates intrinsic structural plasticity of COMT.

**Figure 3:**
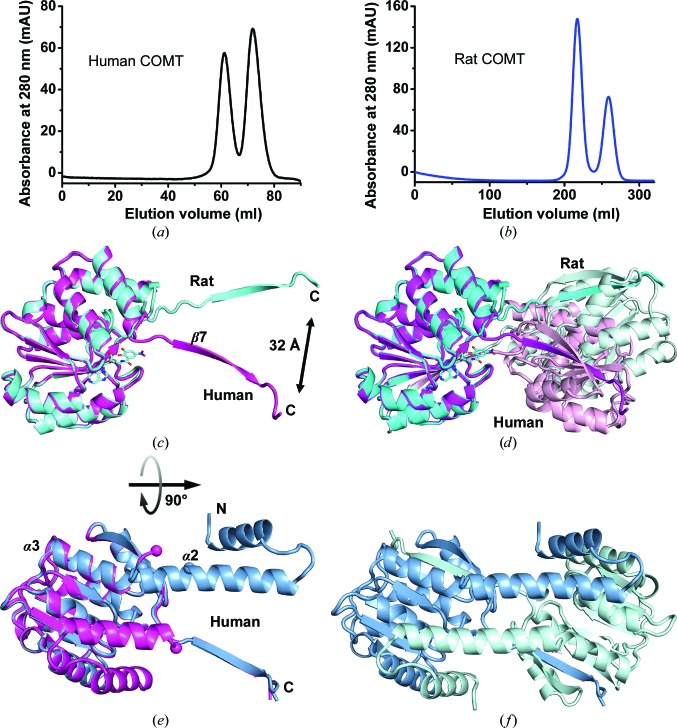
Domain swapping in COMT. (*a*) Gel-permeation chromatography of human COMT on a Superdex S75 16/60 column shows peaks for monomeric and dimeric COMT. Human COMT was placed into the same buffer as rat COMT (see below), or sometimes in 50 m*M* HEPES–NaOH pH 7.0, 150 m*M* NaCl, 2 m*M* MgCl_2_, 1 m*M* TCEP, 10% glycerol. The buffer has no noticeable influence on the monomer:dimer ratio. (*b*) The same experiment for rat COMT on a larger column (home-made S75 26/80), resulting in better separation of the species. The buffer was 50 m*M* Tris–HCl pH 7.5, 50 m*M* NaCl, 10 m*M* DTT, 2 m*M* MgCl_2_. The methyltransferase activities do not differ appreciably between monomer and dimer preparations (data not shown). (*c*) Superposition of rat (cyan; PDB entry 3oe4) and human [magenta; structure (2)] structures that display a swap of their C-terminal β7 strand. The bisubstrate inhibitor in 3oe4 is drawn as a stick model. Its nitrocatechol part would clash with the conformation of the human apo COMT structure, offering an explanation for the discrepancy of the β7 vectors. (*d*) The resulting dimers (lighter hues) therefore show a very different juxtaposition of the monomers when rat and human COMT are compared. (*e*) Example of a double domain swap of human apo COMT (3). In addition to the C-terminal β7 strand, the first 40 residues encompassing helices α1 and α2 have swapped [light blue; structure (3)]. Structure (2) is superimposed as a reference. The disordered α2/α3 loop between Lys86 and Gly93 in structure (2) (human numbering) is marked with spheres. This loop forms part of the α2 helix in structure (3), where these residue positions are also marked. The view in (*e*) is rotated 90° compared with that in (*c*). (*f*) The dimer formed by the double domain swap.

During the purification of rat and human COMT, monomers and dimers were observed for both proteins by gel-permeation chromatography (Figs. 3 ▶ *a* and 3 ▶ *b*). Analytical ultracentrifugation revealed that depending on the COMT preparation 6–32% of rat COMT and 6–46% of human COMT formed dimers, while no higher oligomers were detected (data not shown). Interestingly, a re-analysis of the dimers by gel-permeation chromatography revealed an equilibrium with monomers that could be accelerated by increased temperature (data not shown). The activation energy for the monomer–dimer transition must therefore be rather small, which also explains the large variations in dimer content that was observed for different protein preparations (Figs. 3 ▶ *a* and 3 ▶ *b*).

Several instances of dimer formation by domain swapping in the crystal have previously been observed for ligand-bound rat COMT (Ellermann *et al.*, 2011 ▶). In the rat COMT structures in complex with bisubstrate-analogue inhibitors (PDB entries 3oe4, 3oe5, 3ozs and 3ozt), the C-terminal β-strand β7 swaps to another COMT molecule in the crystal to form a homodimer (cyan in Fig. 3 ▶ *c*). Although only the monomeric forms were used for crystallization, a similar swap of the C-terminal β-strand is visible for human apo COMT (2). The direction of the β7 vector is quite different from that observed in ligand-bound rat COMT, leading to a distance of 32 Å between the C-termini (Fig. 3 ▶ *c*). As a consequence, the associated dimers have very different juxtapositions of their constituent monomers (Fig. 3 ▶ *d*). The reason for the different directions of the swapped strand is likely to be the presence of a ligand at the Mg^2+^ site in the rat COMT structures, which would clash with the protein conformation in human apo COMT (2). This indicates that the β6/β7 hinge region is flexible, in accord with the above comparison with human apo COMT (1). However, neither the rat COMT β6/β7 sequence MKVV nor the human COMT sequence REVV (Fig. 1 ▶ *c*) suggests a high propensity for domain swapping. Such propensity has been ascribed to Gln-, Ala-, Pro- and Gly-rich sequences (Rousseau *et al.*, 2012 ▶), which does not apply to the β6/β7 loop sequences in rat and human COMT. The structural factors that render the β7 strand flexible remain unknown at present.

In another human COMT structure (3) the first 40 N-terminal residues are swapped in addition to the C-terminal β-strand, leading to a doubly domain-swapped dimer (Figs. 3 ▶ *e* and 3 ▶ *f*). A consequence of the swap is an extension of helix α2 and its co-axial alignment with helix α3. The hinge enabling the swap is located at the conserved Gly93, which is consistent with the observed sequence propensity for swapped structures (Rousseau *et al.*, 2012 ▶). It should be noted that structures (2) and (3) are isomorphous (Supplementary Table S2) and in structure (2) the α2/α3 loop region, *i.e.* the linker region for the N-terminal swap, is disordered (Fig. 3 ▶ *e*), indicating that a mixture of singly and doubly swapped molecules may be present in this crystal.

The membrane-bound variant of COMT is a single-pass type II membrane protein with an N-terminal α-helix anchoring the catalytic domain in the membrane. Thus, one would expect biologically relevant dimers to have their N-termini parallel to each other, which is not the case for structures (2) and (3) or any other instance of crystallographic dimer formation observed here [an antiparallel β-sheet extension *via* β7 in structures (4), (5), (6) and (12), not shown]. N- and C-terminal extensions frequently serve to stabilize assemblies ranging from dimers to fibrils, and there might be a link between protein plasticity and the ability to swap out domains or parts of sequences. For example, in the *Borna disease virus* nucleoprotein a planar homotetramer is formed by a swap of both termini (Rudolph *et al.*, 2003 ▶). Among the many examples of domain-swapped structures (reviewed in Rousseau *et al.*, 2012 ▶), RNAse A appears to harbour particular plasticity, forming dimers (Liu *et al.*, 2001 ▶) and trimers (Liu *et al.*, 2002 ▶) by a C-terminal domain swap, dimers *via* an N-terminal domain swap (Merlino *et al.*, 2009 ▶; Liu *et al.*, 1998 ▶) or even fibrils after engineering of a poly-Gln insertion in a hinge loop (Sambashivan *et al.*, 2005 ▶). Double domain swaps have rarely been observed. Examples are the immuno­globulin-binding domain B1 of streptococcal protein G (Frank *et al.*, 2002 ▶) and *Escherichia coli* trp repressor (Lawson *et al.*, 2004 ▶). In conclusion, the domain-swapped dimers of COMT testify to the significant intrinsic plasticity of the enzyme. This plasticity becomes even more apparent when apo structures and mimics of catalytic intermediates are compared. Indeed, the hinge regions β6/β7 and α2/α3 that are critical for the domain swaps form two of the three loops (with the third being β5/α9) that drastically change their conformations upon ligand binding.

### 3.3. Large conformational changes in the active site   

**Figure 4:**
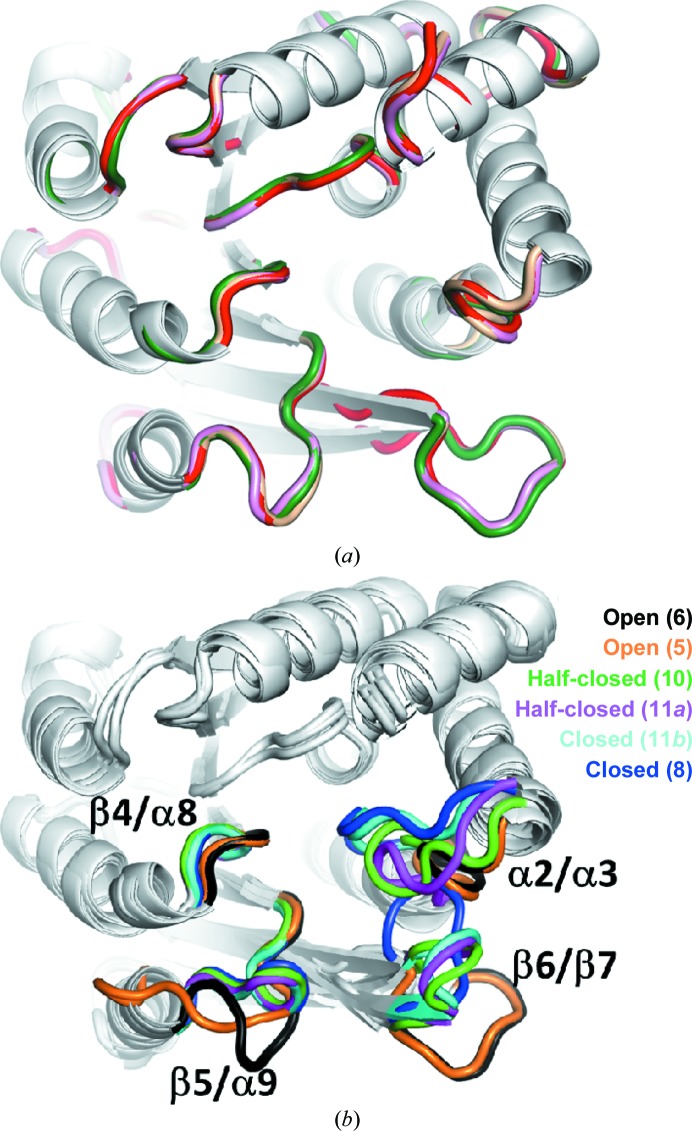
Comparison of rodent apo COMT structures and overall conformational changes of rat COMT upon substrate binding. (*a*) Apo COMT structures from mouse (4) and rat (5, 6) adopt the same conformation. Mouse COMT is marked with green loops. The rat structure (5) contains four molecules in the asymmetric unit, two of which (wheat and pink) have the same conformation as mouse COMT (4). Another rat structure (6) in a different crystal form also shows the same conformation (red). The view is rotated 90° clockwise about the *y* axis with respect to Fig. 2 ▶. (*b*) Loop closure upon substrate binding to rat COMT. No ligands are shown for clarity and the loops that move most are coloured the same for each structure. Structure (6) (black) adopts the most open conformation, followed by (5), (10), the two conformations of (11) and structure (8), which are coloured orange, green, magenta, cyan and blue, respectively. Structure (11) contains two molecules per asymmetric unit that differ in their active-site loop conformations. Structures (7) and (9) have the same conformation as (10) (only the latter is shown)

The apo COMT structures from mouse (4), rat (5, 6) (and the isomorphous structure of Tsuji *et al.*, 2009 ▶) have the same conformation (Fig. 4 ▶ *a*). The conformation of the β6/β7 loop is dominated by crystal contacts, but those of α2/α3 and β5/α9 are not. The different sequences, crystallization conditions and crystal settings (Supplementary Table S1) all indicate that this form is a recurring state of apo COMT. A previously determined rat COMT apo structure (Tsuji *et al.*, 2009 ▶) is isomorphous to structure (6) and shows the same overall fold (r.m.s.d. of 0.5 Å) and side-chain conformations (Fig. 4 ▶ *a*). By contrast, human apo COMT (1) has a very different loop structure around the active site (Fig. 2 ▶), indicating that among the COMT isoforms many more conformations for the apo state might exist that have not yet been captured in crystalline forms. The plasticity of the active-site loops is evident from a comparison of rat COMT structures (Fig. 4 ▶ *b*). Ligand binding induces the loops to come together to form the Mg^2+^-binding site. Side-chain movements and comparatively smaller conformational changes of the β4/α8 loop are required to build the adenine site.

#### 3.3.1. Conformational changes in the adenine site   

**Figure 5:**
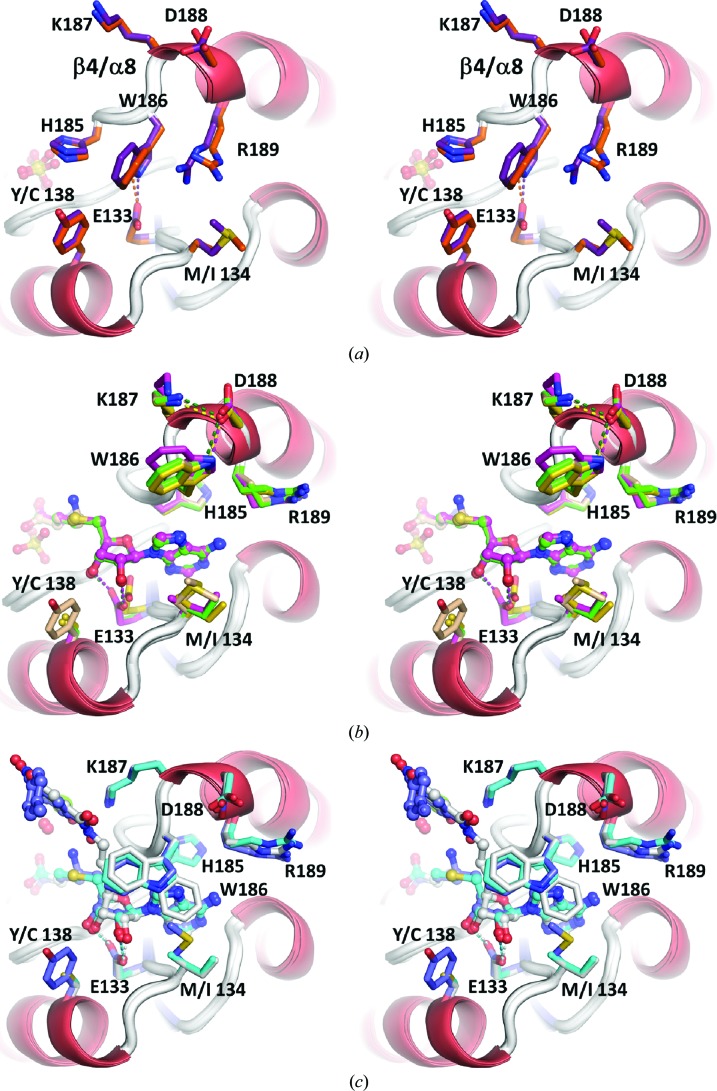
Stereoviews of conformational changes in the adenine site of rat COMT. (*a*) The two apo COMT structures (5) (orange) and (6) (violet) and a previously determined rat COMT structure (grey; PDB entry 2zlb; Tsuji *et al.*, 2009 ▶) have the same side-chain conformations in the adenine site. The structures have phosphate or sulfate bound at the site where the carboxylate group of SAM normally binds. Trp186 occupies roughly the usual position of the adenine base and hydrogen-bonds to Glu133, which normally binds the ribose hydroxyl groups. His185 is swung out by 7.4 Å (C^γ^ atom distance) relative to its position when substrate is bound and packs perpendicularly onto the indole ring of Trp186. The other side of the indole ring is contacted by Arg189. (*b*) The half-closed conformation is adopted by four COMT structures: (7) (sinefungin, wheat), (9) (sulfate, yellow), (10) (SAH, green) and (11*a*) (SAH, magenta). Asp188 hydrogen-bonds to the indole N atom of Trp186, keeping it from packing on top of the adenine base. Lys187 is in the out-conformation away from the Mg^2+^ site and interacts electrostatically with Asp188. His185 has swung over to the other side and is buried under the adenine base in a previously observed perpendicular interaction of the aromatic groups. Also, Arg189 has swung out of the adenine site. Structure (9) is notable because the half-closed conformation is adopted in the absence of a compound in the adenine site. (*c*) The closed conformation is adopted by two novel COMT structures (8) (sinefungin/tolcapone, blue) and (11*b*) (SAH, cyan). This is the standard conformation of substrate-bound COMT that is also adopted in bisubstrate-inhibitor complexes. As a reference PDB entry 3oe4 (Ellermann *et al.*, 2011 ▶) is shown (grey). Asp188 adopts another rotamer and releases both Lys187 and Trp186. Trp186 now packs perpendicularly on top of the adenine base and the catalytic Lys187 is in the in-conformation towards the Mg^2+^ site.

The set of crystal structures described here can be divided into three overall classes of adenine-site conformations: an open or collapsed state (Fig. 5 ▶ *a*), a half-closed state (Fig. 5 ▶ *b*) and a closed state (Fig. 5 ▶ *c*). It will become apparent that adoption of the half-closed state does not depend on the presence of a ligand in the adenine-binding site.

In the open state neither the adenine site nor the Mg^2+^ site are formed. Residues His185, Trp186 and Arg189 stack on top of each other, with Trp186 occupying the usual position of the adenine base. In the absence of a ribose, Glu133 hydrogen-bonds to the indole N atom of Trp186. The largest conformational change compared with the closed conformation (see below) is undergone by His185, which swings out by ∼7.4 Å relative to its position when substrate is bound.

In the half-closed state [structures (7), (9), (10) and one protomer of (11)] some, but not all, side chains have moved into the position in the closed state (Fig. 5 ▶ *b*). His185 in the β4/α8 loop has swung underneath the adenine base and packs against the purine ring, which has displaced Trp186 and Arg189 from the binding pocket. As soon as a ribose is present (from SAH or sinefungin) the carboxylate of Glu133 forms two hydrogen bonds to the 2′- and 3′-hydroxyl groups. Interestingly, in all four structures Lys187 now electrostatically contacts or even hydrogen-bonds to Asp188 in a so-called out-conformation away from the Mg^2+^ site (see also below). This interaction is absent in the open and closed forms (Figs. 5 ▶ *a* and 5 ▶ *c*). Another hydrogen bond is formed between the side chains of Asp188 and Trp186, fixing the indole in a conformation away from the adenine base. Importantly, this concerted movement of side chains is not strictly dependent on the presence of an adenine-containing ligand since in structure (9) the half-closed conformation is adopted in the absence of a compound in the adenine site.

Few conformational changes are now required to reach the closed state and to complete formation of the adenine pocket. The structure of the rat COMT–sinefungin–tolcapone complex (8) represents the closed Michaelis state and is almost identical to the previously described complex of rat COMT–SAM–tolcapone (PDB entry 3s68; Ellermann *et al.*, 2012 ▶). The closed state currently represents the best studied conformation of COMT (Ellermann *et al.*, 2009 ▶, 2011 ▶, 2012 ▶; Rutherford *et al.*, 2008 ▶; Palma *et al.*, 2006 ▶; Bonifácio *et al.*, 2002 ▶; Lerner *et al.*, 2001 ▶; Vidgren *et al.*, 1994 ▶). Compared with the half-closed state, two residues change side-chain conformation: Trp186 is released from Asp188 and now packs in an edge-to-face fashion onto the adenine base (Fig. 5 ▶ *c*). Lys187 also is released from Asp188 and adopts the so-called in-conformation, pointing towards the Mg^2+^ site. Compared with the adenine site, the formation of the Mg^2+^ site requires much more drastic conformational changes as described in the following.

#### 3.3.2. Conformational changes in the Mg^2+^ site   

**Figure 6:**
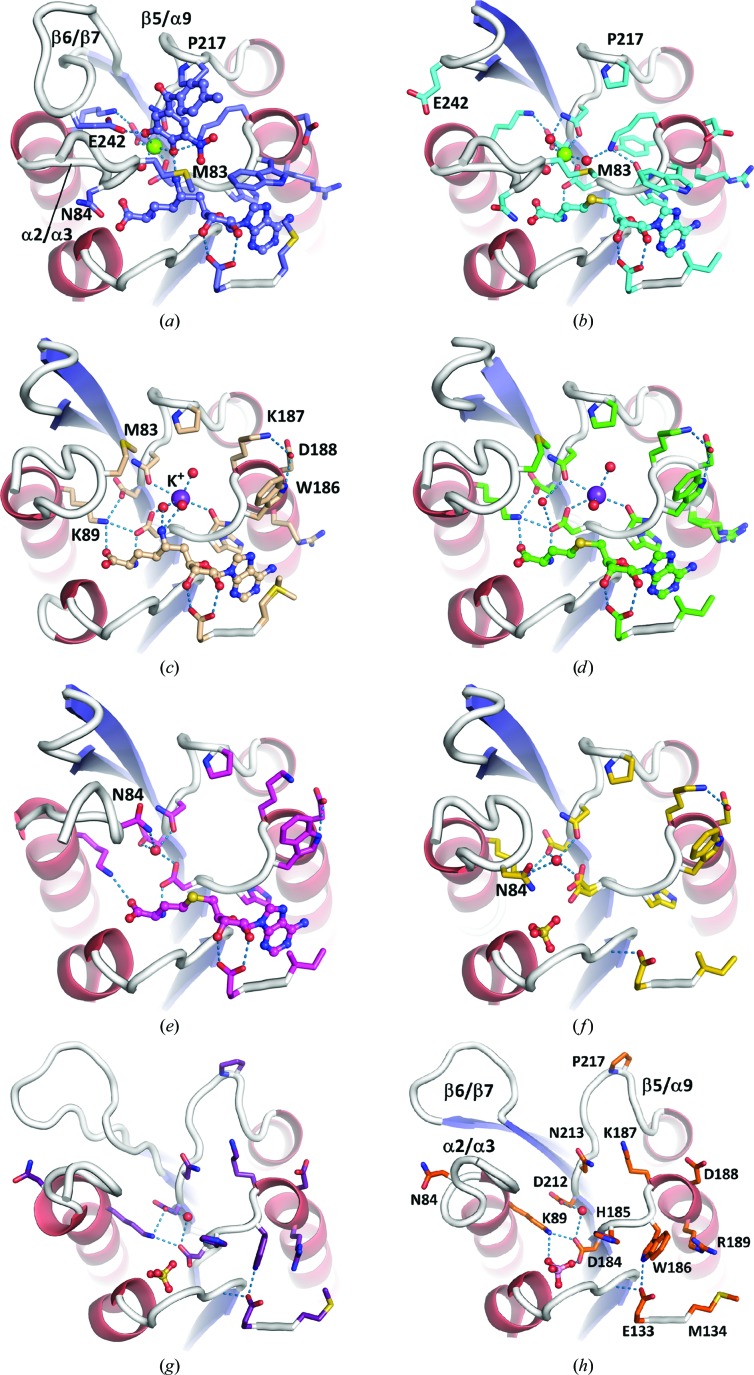
Conformational changes in the Mg^2+^-binding site. This figure recapitulates some of the information in Fig. 5 ▶ from a different angle while focusing on the Mg^2+^ site. (*a*) and (*b*) show the closed state of COMT with an octahedral coordination of the Mg^2+^ ion (green sphere). Lys187 is in the in-conformation. (*c*)–(*f*) show the half-closed conformation with or without a ligand (SAH or sinefungin) in the adenine site. Lys187 is in the out-conformation. (*g*) and (*h*) are the open conformations where neither substrate-binding site is formed. Lys187 is in an intermediate conformation. (*a*) Structure (8) with sinefungin and tolcapone represents the Michaelis complex with SAM replaced by the inhibitor sinefungin and the substrate catechol replaced by the inhibitor tolcapone. (*b*) (11*b*) has Mg^2+^ bound but instead of a catechol two water molecules complete the coordination sphere of Mg^2+^. (*c*) The sinefungin-bound structure (7) lacks the Mg^2+^ ion but has a K^+^ ion bound to a nearby site. K^+^ coordinates the former Mg^2+^-binding side chains Asp184 and Asn213. The primary ammonium group of sinefungin bridges *via* a water molecule to the K^+^ ion. Lys89, which normally neutralizes the charge of Asp212, reorients towards the carboxylate of SAH. (*d*) Structure (10) bound to SAH recapitulates all of the salient features of (*c*). (*e*) (11*a*) bound to SAH has no K^+^ ion bound but retains most of the side-chain positions with the exception of Asn84 in the α2/α3 loop, which moves to contact a water molecule situated at the former position of the Mg^2+^ ion. (*f*) Structure (9) is interesting as it adopts the same half-closed conformation as (7), (10) and (11*a*) in the absence of a ligand in the adenine site. As in (*e*), water replaces Mg^2+^. Sulfate is located at the site where the carboxylate of SAM and related compounds normally binds. In the absence of a ribose, Glu133 reorients to hydrogen-bond to the main-chain amide N atom of Ala110. (*g*) The apo COMT structure (6) also does not have a functional Mg^2+^ site and water hydrogen-bonds to Asp184 and Asp212. Pro217 in the β5/α9 loop, which normally engages in van der Waals contact with the aryl part of the catechol substrate, has maximally swung away from the body of COMT. (*h*) Structure (5) has the same overall conformation as structure (6) in (*g*).

In the rat COMT–sinefungin–tolcapone complex (8) the Mg^2+^ ion is octahedrally coordinated by the catechol, a water molecule and the side chains of Asp184, Asp212 and Asn213 (Fig. 6 ▶ *a*). This structure should closely resemble the Michaelis complex, in which SAM is replaced by the inhibitor sinefungin and tolcapone serves as an unreactive catechol substrate analogue. With an r.m.s.d. of 0.25 Å, structure (8) is indeed virtually indistinguishable from the COMT–SAM–tolcapone complex (PDB entry 3s68; Ellermann *et al.*, 2012 ▶), affirming that sinefungin is a valid SAM analogue. Loop β5/α9 has folded towards the Mg^2+^ site, placing the pyrrole side chain of Pro217 in van der Waals contact with the catechol (Fig. 6 ▶ *a*). Loops β6/β7 and α2/α3 cover the Mg^2+^ site. Glu242 in β6/β7 binds to the catechol hydroxyl group that is not modified (labelled 2 in Figs. 1 ▶ *a* and 1 ▶ *b*) and the hydrophobic side chain of Met83 in α2/α3 packs onto the aromatic part of the catechol (Fig. 6 ▶ *a*).

From all previous COMT crystal structures it appeared that the binding of Mg^2+^ strictly requires the presence of catechol. However, a holo form of COMT without catechol can exist, as represented by (11*b*), in which two water molecules occupy the coordination sites of the catechol (Fig. 6 ▶ *b*). Despite the absence of a catechol, the hydrophobic residues Met83 and Pro217, which previously sandwiched the catechol, barely move. In contrast, Glu242 in β6/β7 flips away from the Mg^2+^ site by 6 Å (C^α^–C^α^ distance) and does not bind to the water molecules that replace the catechol. The closed holo structure (11*b*) testifies to the ordered bi-bi mechanism described for COMT, which assumes that SAM binds first followed by Mg^2+^ and the catechol substrate (Tunnicliff & Ngo, 1983 ▶; Vidgren *et al.*, 1994 ▶; Lotta *et al.*, 1995 ▶). It is probably safe to assume that the reverse process holds for product release, *i.e.* first the methylated catechol leaves, followed by Mg^2+^ and SAH. In this case (11*b*) represents a true catalytic intermediate and the holo complex for the forward reaction can be modelled simply by adding a methyl group to SAH *in silico*.

Following the release of Mg^2+^, a conformational change in loop α2/α3 removes Met83 from the Mg^2+^ site while Pro217 in loop β5/α9 stays put. This half-closed state is trapped in the isomorphous structures (7) and (10), which have sinefungin and SAH bound at the adenine site, respectively (Figs. 6 ▶ *c* and 6 ▶ *d*). The surplus of negative charge after the departure of Mg^2+^ is partially neutralized by Lys89, which changes conformation relative to the closed conformation and now binds to both Asp184 and Asp212. In addition, a K^+^ ion from the crystallization medium is recruited to a site close to the former Mg^2+^ site. K^+^ is bound by Asp184 and Asn213, the side chains of which have moved slightly after the release of Mg^2+^. Interestingly, Lys187 is flipped out of the Mg^2+^ site and now binds to Asp188. The K^+^ ion in (7) and (10) binds at the same position as vacated by the ammonium group of Lys187 when switching from the in-conformation to the out-conformation. However, a cation is not necessary for maintaining the half-closed conformation. In the SAH-bound structure (11*b*) and in the apo structure (9) the absence of a cation has little effect on side-chain conformations, but a water molecule is recruited to the Mg^2+^ site (Figs. 6 ▶ *e* and 6 ▶ *f*). The exception is Asn84 in loop α2/α3 of (9) and (11), which moves by 4–6 Å relative to the closed conformation to contact the Mg^2+^-replacing water molecule. Notably, neither a ligand in the Mg^2+^ site nor a ligand in the adenine site is required for COMT to adopt the half-closed conformation, as exemplified by the apo structure (9) (Fig. 6 ▶ *f*). A sulfate is located at the site where the carboxylate of SAM normally binds, but tetrahedral anions have no influence on COMT conformation since sulfate and phosphate are also present in the open conformations (6) and (5) (Figs. 6 ▶ *g* and 6 ▶ *h*, respectively). These apo COMT structures have neither functional Mg^2+^ nor functional adenine sites, but share a similar overall conformation. Water replaces Mg^2+^ and is contacted by Asp184 and Asp212. The β5/α9 loop has now swung out to its maximum extent.

Taken together, the above-described array of structures illustrates the stepwise transition from the closed to the open conformation of COMT. Similar conformations are adopted more than once in independent structures, indicating that they are energetically favourable and should be true intermediates along the reaction coordinate. Some of the conformational changes observed in the apo structures yield insight into the catalytic details of methyl transfer.

### 3.4. Mechanistic implications for methyl transfer   

In principle, COMT catalyses a simple S_N_2 transfer of an electrophilic methyl group from SAM onto a nucleophilic O atom of a catechol (labelled 1 in Figs. 1 ▶ *a* and 1 ▶ *b*). The transfer is facilitated by in-line juxtaposition of the reactants and by lowering of the p*K*_a_ of the catechol upon binding to the Lewis acid Mg^2+^ (Vidgren *et al.*, 1994 ▶). However, it is not entirely clear from the many high-resolution COMT crystal structures whether the catechol is deprotonated when ligated to Mg^2+^. There is no example of a catechol–Mg^2+^ complex crystal structure in the Cambridge Structural Database that would reveal H atoms, and we were hitherto unsuccessful in crystallizing a 2,4-dinitrocatechol–Mg^2+^ complex at physio­logical pH. It has been suggested (Vidgren *et al.*, 1994 ▶) that the proton on the other hydroxyl group of the catechol (labelled 2 in Figs. 1 ▶ *a* and 1 ▶ *b*) is stabilized by interaction with the carboxylate of Glu242 (Fig. 6 ▶ *a*). From hydrogen-bonding considerations it is unclear whether the apically bound solvent molecule is indeed a water or a hydroxide, rendering charge-neutralization calculation difficult. Given that Mg^2+^ is a comparatively weak Lewis acid (stronger than Ca^2+^ but much weaker than, for example, Fe^2+^ or Al^3+^), a role of Lys187 acting as a Brønsted base during catalysis might be envisaged. The fact that Lys187 can flip in and out of the Mg^2+^ site (Figs. 5 ▶ and 6 ▶) enables facile proton exchange with bulk solvent. Such a function may not be needed for stronger Lewis acids of comparable ionic radius such as Mn^2+^, Co^2+^, Zn^2+^ and Fe^2+^, which can functionally replace Mg^2+^ (Axelrod & Tomchick, 1958 ▶). Indeed, Mn^2+^ and Co^2+^ even lead to increased COMT activity compared with Mg^2+^ (Axelrod & Tomchick, 1958 ▶). In these complexes, acidification of the catechol hydroxyl by the metal ion is likely to be sufficient for generation of the phenolate nucleophile. However, for the physiologically relevant weaker Lewis acid Mg^2+^ the possible catalytic importance of the Lys187 in-conformation now becomes clear since its terminal amino group is at a hydrogen-bonding distance from the hydroxyl group 1 that will be methylated (Fig. 6 ▶ *a*). The positive charge of Mg^2+^ might also decrease the p*K*_a_ of the Lys187 side chain since juxtaposition of an ammonium group next to the metal ion [3.8 Å distance in (8)] would result in Coulomb repulsion. Thus, after abstraction of a proton from the substrate [2.8 Å distance from the catechol hydroxyl group 1 in (8)], the ammonium group of Lys187 should be expelled from the catalytic site while at the same time nucleophilic attack of the phenolate on SAM occurs. Another aspect of COMT catalysis relates to the acidity of the catechol hydroxyl group and the polarizability (and hence nucleophilicity) of the resulting phenolate. Nitro groups in catechols reduce the p*K*_a_ values of the hydroxyl groups to such an extent that the phenolates are bound tightly to Mg^2+^ and are unreactive towards SAM (for example, tolcapone and 2,4-dinitro­catechol). The Lewis acidity of Mg^2+^ must be low enough to not render the substrate phenolate inactive but within a range to allow, possibly in conjunction with Lys187, the generation of a nucleophile. By contrast, very strong Lewis acids such as Fe^3+^ and Al^3+^ abolish COMT activity (Axelrod & Tomchick, 1958 ▶), possibly by the same mechanism as do nitro groups: the high acidity of the hydroxyl group when bound to the metal ion results in a less polarisable phenolate of low nucleophilicity.

It has been suggested that after methyl transfer has occurred, Lys187 repels the methyl group of the *O*-methyl ether product from the active site (Vidgren *et al.*, 1994 ▶). While this is true for the in-conformation of Lys187, several examples (Figs. 5 ▶ *a*, 5 ▶ *b* and 6 ▶ *c*–6 ▶ *h*) show Lys187 in the out-conformation, indicating facile transition between these states. Thus, the driving force for expelling the product is unlikely to come from van der Waals repulsion by Lys187, which itself is mobile. Rather, the *O*-methyl ether is a poor Lewis base and will bind much less strongly to the Mg^2+^ ion than the phenolate (or hydroxyl) of a catechol substrate. Thus, methylation conveniently leads to product release from the metal ion. Since Asp184 bridges the ammonium group of SAH and Mg^2+^ (Fig. 6 ▶ *b*), release of the co-product SAH should automatically lead to destruction of the Mg^2+^ site. The Mg^2+^ site then has to be re-ordered after SAM has bound, possibly into the holo structure (11*b*; Tunnicliff & Ngo, 1983 ▶; Vidgren *et al.*, 1994 ▶; Lotta *et al.*, 1995 ▶). The strict involvement of a lysine in COMT catalysis is challenged by the sequence of a novel variant (COMT2; Supplementary Fig. S2) which has a proline instead of Lys187 (Du *et al.*, 2008 ▶). In addition, the two Mg^2+^-binding residues Asn170 and Asp184 in COMT are replaced by a histidine and an alanine, respectively, in COMT2. Under the assumption that COMT2 still binds Mg^2+^, a homology model based on the closed Michaelis-type conformation (8) revealed that histidine could functionally replace lysine as a catalytic base (data not shown).

The poor catalytic efficiency of 24 min^−1^ for rat liver COMT (Schultz & Nissinen, 1989 ▶) raises the question of what the rate-limiting step of the reaction might be. Candidates are slow substrate binding or slow dissociation of products, possibly limited by conformational changes of COMT. The co-substrate SAM has a *K*_m_ value of 40–60 µ*M* for soluble COMT (Coward *et al.*, 1972 ▶; Lotta *et al.*, 1995 ▶) and SAH inhibits COMT with an IC_50_ of ∼30 µ*M* (Coward *et al.*, 1972 ▶). The *K*_d_ value for SAM was determined to be 20 µ*M* (Lotta *et al.*, 1995 ▶). Such weak affinities are not unusual for metabolites and often indicate high off-rates, which would exclude product dissociation as rate-determining. Even with a hypothetical and relatively slow *k*_on_ of 10^4^ *M* ^−1^ s^−1^ for SAM or SAH, the relation *K*_d_ = *k*_off_/*k*_on_ would return a *k*_off_ value of 5 s^−1^ (300 min^−1^), which is still larger than the catalytic efficiency. The association of small molecules with proteins that do not undergo large conformational changes should also not be rate-limiting. Based on the crystal structure in the closed form, a conformational change was hypothesized early on that could facilitate SAH dissociation (Vidgren *et al.*, 1994 ▶). The apo crystal structures presented here reveal an array of conformational changes for three loops that come together to form the bipartite active site. This concerted movement itself might impose a rate limitation on catalysis, but more experiments are needed to shed light on this issue.

### 3.5. Apo COMT as a possible drug target   

Despite numerous interactions with COMT, the *K*_m_ value of SAM is large, indicating that much (but not all) of its binding energy is either counteracted by desolvation or protein conformational changes to shape the active site. In addition, binding energy may be converted into potential energy within COMT that facilitates the liberation of reaction products, a general feature that is often encountered in enzymes (Jencks, 1987 ▶). In order to circumvent unnecessary enthalpic or entropic loss of binding energy, a COMT inhibitor that acts on the apo state might be an option. As the apo state is an obligatory intermediate during the catalytic cycle, such an inhibitor could lock COMT in an unresponsive conformation after a single round of catalysis, although it would need to compete with presumably high intracellular concentrations of SAM. Since an apo-state inhibitor does not have to compete with molecules in the active site, it has the potential advantages of a very different chemical composition and improved physicochemical properties. Here, a surface-binding ureido-benzamidine is discussed that acts like a lid on top of the collapsed adenine-binding pocket and may serve as a starting point for the design of inhibitors of apo COMT.

**Figure 7:**
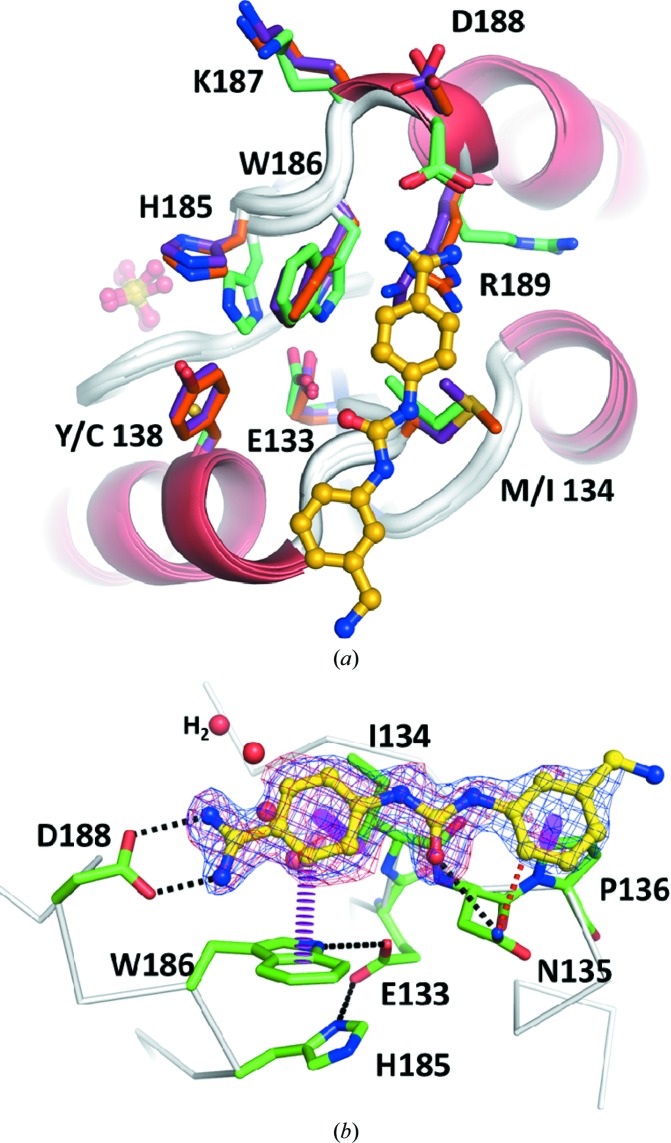
Binding of the inhibitor 4-[3-(3-aminomethylphenyl)-ureido]-benzamidine to the surface of humanized rat apo COMT. (*a*) Superposition of the apo COMT structures (5) and (6) with the complex structure (12) (green). The orientation is the same as in Fig. 5 ▶ and shows two notable side-chain differences for His185 and Arg189 in the complex structure. (*b*) The 2*mF*_o_ − *DF*_c_ electron-density map for the ligand is contoured at 1.5 r.m.s.d. (blue). The *mF*_o_ − *DF*_c_ OMIT map is contoured at 2.5 r.m.s.d. (red). From the densities it is apparent that the benzamidine moiety is the best ordered part of the ligand. The positively charged head group forms two hydrogen bonds to Asp188. Another hydrogen bond is formed between the carbonyl group of the urea moiety and Asn135. The two aromatic parts of the inhibitor are in van der Waals contact with Trp186, Ile134 and Pro136. A close contact between the side chain of Asn135 and the inhibitor may explain the relatively low affinity of the compound. A water-filled cavity (red spheres) is cordoned off by the inhibitor, leaving options for inhibitor improvement by water displacement. Although the structure is with humanized rat COMT, all residues that contact the inhibitor are conserved in human COMT and the binding mode will thus be the same for both forms. Hydrogen bonds, van der Waals interactions and clashes are shown as black, magenta and red dashed lines, respectively.

A high-throughput screen for COMT inhibitors yielded a weakly binding ureido-benzamidine derivative (IC_50_ = 4.4 µ*M*). The co-crystal structure of this compound in complex with humanized rat COMT (12) was determined to a resolution of 1.4 Å with two molecules in the asymmetric unit that both bind to the inhibitor in the same fashion. The molecule is bound on top of the adenine site at the surface of COMT (Fig. 7 ▶ *a*). The adenine site is collapsed similarly as in the apo COMT structures (5) and (6): Trp186, which normally packs on top of the adenine base of SAM (Fig. 5 ▶ *c*), now occupies the adenine pocket. His185 packs against Trp186 in a roughly parallel orientation, in contrast to the usual perpendicular orientation of His185 and adenine when SAM or a related molecule is bound. Glu133, which normally binds to the ribose hydroxyl groups in SAM, bridges His185 and Trp186 *via* two hydrogen bonds. This COMT conformation is similar to the apo form represented by structures (5) and (6) (Fig. 5 ▶ *c*), but also exhibits significant differences. For instance, in (5) and (6) the His185/Trp186 side-chain interaction is perpendicular, but it is almost parallel in (12). Also, Glu133 acts as a clamp by binding to both His185 and Trp186, while in (5) and (6) only the indole of Trp186 is bound. Lastly, Arg189 contacts the Trp186 indole in structures (5) and (6) but points into the solvent in (12). The cavity left behind is filled with solvent (see below).

The inhibitor forms three possible hydrogen bonds to COMT and shields the hydrophobic Ile134, Pro136 and part of Trp186 from solvent. A close contact is present with the side chain of Asn135 that may explain the rather low affinity of the inhibitor. The inhibitor cordons off a solvent-filled cavity (Fig. 7 ▶ *b*). Removing the clash, *e.g.* by substituting a pyridine for the benzene ring involved, and extending the inhibitor into the solvent-filled cavity are possible routes for inhibitor improvement. The mode of interaction on the COMT surface is suboptimal since competition of the inhibitor with bulk solvent for hydrogen-bonding partners will cost binding energy. In addition, the positive charge introduced by the basic amidine should be avoided if membrane penetration, especially into the brain for the treatment of schizophrenia, is desired. This problem could be addressed by grafting known head groups from aspartic protease inhibitors such as β-secretase I. An amidine forming a bidentate hydrogen bond with an aspartate of β-secretase I has been replaced by an array of 25 different head groups, among which were aminothiazines, 2-aminooxazolines, aminohydantoins, 2-aminodi­hydropyrimidinones and aminopiperidines (Woltering *et al.*, 2013 ▶). The situation is the same in the COMT structure (12), and the same head groups might be used to replace the amidine of the current inhibitor. These partially saturated head groups have improved physicochemical properties with respect to solubility, permeability, log*D* and p*K*_a_ compared with an amidine, and additionally provide exit vectors for substituents that can reach into the water-filled pocket (Fig. 7 ▶ *b*), thereby potentially contributing both affinity and selectivity for COMT.

## 4. Conclusions and outlook   

Comparison of COMT structures in several crystal forms and in several conformational states has revealed a set of three loop regions that come together when the Michaelis complex is formed. While crystal contacts that possibly trap conformations that are not populated in solution are always a concern, the three classes of conformations (closed, half-closed and open) that were observed several times under different crystallographic circumstances lend credibility to the notion that they constitute *bona fide* catalytic intermediates. Each of these conformations is a drug target, offering the possibility of finding inhibitors with novel binding modes and novel inhibition mechanisms. A single apo-state inhibitor was discovered during an activity-based high-throughput screen of COMT. Most of the current COMT inhibitors bind to the Mg^2+^ ion by way of a catechol. However, catechols and related acidic metal-binding moieties are often subject to oxidation, for example, by cytochromes (Haining & Nichols-Haining, 2007 ▶) or xanthine oxidase (Foppoli *et al.*, 1997 ▶). They also suffer from efficient excretion after glucuronidation (Antonio *et al.*, 2002 ▶) and do not penetrate membranes well. An apo COMT inhibitor thus holds promise for the development of ways of modulating neurotransmitter concentrations in the schizophrenic brain.

## 5. Related literature   

The following references are cited in the Supporting Information: Diederichs & Karplus (1997 ▶), Karplus & Diederichs (2012 ▶) and Winn *et al.* (2011 ▶). 

## Supplementary Material

Supporting Information.. DOI: 10.1107/S1399004714012917/yt5071sup1.pdf

PDB reference: catechol-*O*-methyltransferase, 4pyk

PDB reference: 4pyq

PDB reference: 4pym

PDB reference: 4pyj

PDB reference: 4p7f

PDB reference: 4pyn

PDB reference: 4pyi

PDB reference: 4p7j

PDB reference: 4p7k

PDB reference: 4pyl

PDB reference: 4pyo

PDB reference: 4p7g

## Figures and Tables

**Table 1:** Summary of crystal structures

No.	Protein	State, ligand(s)	Remarks
(1)	hCOMT	Open, none	First apo structure of human COMT, monomer (Fig. 2 ▶).
(2)	hCOMT	Open, none	Dimer formation by swapping of C-terminal β-strand (Figs. 3 ▶ *c*, 3 ▶ *e*).
(3)	hCOMT	Open, none	In addition to (2), N-terminal 40 residues swapped (Fig. 3 ▶ *e*).
(4)	mCOMT	Open, phosphate	First mouse COMT structure in the apo form, phosphate bound at the site where SAM carboxylate normally binds (Figs. 2 ▶, 4 ▶ *a*).
(5)	rCOMT	Open, phosphate	Apo structure of rat COMT, phosphate bound at the site where SAM carboxylate normally binds. Two of the four protomers have the same conformation as mouse COMT (4) (Figs. 4 ▶ *a*, 4 ▶ *b*, 5 ▶ *a*, 6 ▶ *h*).
(6)	rCOMT	Open, sulfate	Another apo structure, sulfate bound at the site where SAM carboxylate normally binds; quite similar to (5) but a different crystal form. Same overall conformation as (4). Isomorphous to the apo structure described in Tsuji *et al.* (2009 ▶) (Figs. 4 ▶ *a*, 4 ▶ *b*, 5 ▶ *a*, 6 ▶ *g*).
(7)	rCOMT	Half-closed, sinefungin	Co-crystal structure with the SAM analogue sinefungin. Semi-holo form. No Mg^2+^ but K^+^ nearby (Figs. 6 ▶ *b*, 6 ▶ *c*).
(8)	rCOMT	Closed, sinefungin and tolcapone	First structure with the sinefungin/tolcapone ligand combination. Similar to many other closed COMT structures, including those with bisubstrate inhibitors. Solvent-exposed part of tolcapone is disordered (Figs. 4 ▶ *b*, 5 ▶ *c*, 6 ▶ *a*).
(9)	hrCOMT	Half-closed, sulfate	Apo structure with sulfate bound at the site where SAM carboxylate normally binds (Figs. 6 ▶ *b*, 6 ▶ *f*).
(10)	hrCOMT	Half-closed, SAH	Complex with SAH. Semi-holo form. Mg^2+^ site not formed but K^+^ nearby, similar to (7) (Figs. 4 ▶ *b*, 5 ▶ *b*, 6 ▶ *d*).
(11)	hrCOMT	(11*a*), half-closed, SAH; (11*b*), closed, SAH, Mg^2+^	Two very different structures in the same crystal, both bound to SAH. (11*a*) is similar to (9), despite the presence of a large ligand in the adenine site. Semi-holo form. Out-conformation of Lys187 (Figs. 4 ▶ *b*, 5 ▶ *b*, 6 ▶ *e*). (11*b*) Holo form: adenine site is occupied by SAH and Mg^2+^ is present but no catechol substrate is bound. In-conformation of Lys187 (Figs. 4 ▶ *b*, 6 ▶ *b*).
(12)	hrCOMT	Open/ureido-benzamidine	First example of an inhibitor bound to the apo state of COMT (Fig. 7 ▶).

## Footnotes

1 Abbreviations: apo form, no or a simple ligand such as sulfate or phosphate bound to the adenine site of COMT; COMT, catechol-*O*-methyltransferase; (h,m,r)COMT, human, mouse, rat COMT; holo form, COMT with SAM (or a structural analogue such as SAH or sinefungin) and Mg^2+^ bound but no catechol; hrCOMT, humanized rCOMT, *i.e.* rCOMT double mutant M134I/Y138C; Michaelis form, fully assembled COMT, including a catechol bound to Mg^2+^; r.m.s.d., root-mean-square distance; SAH, *S*-adenosylhomocysteine; semi-holo form, COMT with SAM (or a structural analogue) bound but not Mg^2+^; SAM, *S*-adenosylmethionine.

2 Supporting information has been deposited in the IUCr electronic archive (Reference: YT5071).
