# Supplementary material for: Mapping the conformational space accessible to catechol-O-methyltransferase
Source: Acta Crystallogr D Biol Crystallogr. 2014 Jul 25;70(Pt 8):2163–74. doi: 10.1107/S1399004714012917 (PMC4118827; doi:10.1107/S1399004714012917)
Supplement: Supplementary file 1 [file d-70-02163-sup1.pdf]

Acta Crystallographica Section D

Volume 70 (2014)

Supporting information for article:

Mapping the conformational space accessible to catechol-*O*-methyltransferase

Andreas Ehler, Jörg Benz, Daniel Schlatter and Markus G. Rudolph

Table S1 Crystallization conditions

COMT Crystal	Human apo	Human apo<br>single domain swap	human apo<br>double domain swap	Mouse apo<br>Phosphate	Rat apo<br>Phosphate	Rat apo<br>Sulphate
# / PDB-ID	**(1)** / 4PYI	**(2)** / 4PYJ	**(3)** / 4PYK	**(4)** / 4P7F	**(5)** / 4P7G	**(6)** / 4P7J
[Protein]	11.1 mg/ml	13.5 mg/ml	11.9 mg/ml	10.5 mg/ml	13 mg/ml	13mg/ml
Protein buffer	50 mM HEPES/NaOH pH 7.0, 50 mM NaCl, 2 mM DTT, 1 mM EDTA	50 mM HEPES/NaOH pH 7.0, 50 mM NaCl, 2 mM DTT, 1 mM EDTA	50 mM HEPES/NaOH pH 7.0, 50 mM NaCl, 2 mM DTT, 1 mM EDTA	50 mM Tris/HCl pH 7.5, 50 mM NaCl, 10 mM DTT, 2 mM $MgCl_2$	50 mM Tris/HCl pH 7.5, 50 mM NaCl, 10 mM DTT, 2 mM $MgCl_2$	20 mM BisTris/HCl pH 6.2, 100 mM NaCl, 5 mM DTT, 5 mM $MgCl_2$
Ligand	-	-	-	Phosphate [1)]	Phosphate [1)]	Sulphate [1)]
Reservoir	27.5% PEG 3350, 0.1M Tris/HCl pH 8.0, 0.2M $MgCl_2$	22.5% PEG 1500, 0.1M Tris/HCl pH 8.0, 0.2M $MgCl_2$	22.5% PEG 3350, 0.1M Tris/HCl pH 7.5, 0.2M $MgCl_2$	20% PEG 1000, 0.1M Na_2HPO_4-CitricAcid pH 4.2, 0.2M Li_2SO_4	1.6M NaH_2PO_4 / 0.4M K_2HPO_4, 0.1M Na_2HPO_4-CitricAcid pH 4.2	30% PEG 5000 MME, 0.1M MES/NaOH pH 6.5, 0.2M $(NH_4)_2SO_4$
Drop volume / protein content	0.6ul / 75%	0.6ul / 70%	0.6ul / 60%	0.4 ul / 75%	0.4ul / 75%	0.4ul / 50%
Remarks	Seeding	Seeding	Seeding	-	-	-

Crystal	Rat<br>Sinefungin	Rat<br>Sinefungin,<br>Tolcapone	Humanised Rat<br>apo, Sulphate	Humanised Rat<br>SAH	Humanised Rat<br>SAH semi-holo	Humanised Rat<br>ureido-benzamidine
# / PDB-ID	**(7)** / 4P7K	**(8)** / 4PYL	**(9)** / 4PYM	**(10)** / 4PYN	**(11)** / 4PYO	**(12)** / 4PYQ
[Protein]	13 mg/ml	15.5 mg/ml	16 mg/ml	13 mg/ml	16 mg/ml	13 mg/ml
Protein buffer	50 mM Tris/HCl pH 7.5, 50 mM NaCl, 10 mM DTT, 2 mM $MgCl_2$	20 mM BisTris/HCl pH 6.2, 100 mM NaCl, 5 mM DTT, 5 mM $MgCl_2$	50 mM Tris/HCl pH 7.5, 50 mM NaCl, 10 mM DTT, 2 mM $MgCl_2$	50 mM Tris/HCl pH 7.5, 50 mM NaCl, 10 mM DTT, 2 mM $MgCl_2$	50 mM Tris/HCl pH 7.5, 50 mM NaCl, 10 mM DTT, 2 mM $MgCl_2$	50 mM Tris/HCl pH 7.5, 50 mM NaCl, 10 mM DTT, 2 mM $MgCl_2$
Ligand	Sinefungin (50-fold molar excess) [2)]	Sinefungin, Tolcapone [3)]	Sulphate [1)]	SAH (50-fold molar excess) [2)]	SAH (10-fold molar excess) [2]	4 mM ligand + 2% DMSO[4)], SAH (10-fold molar excess) [2)]
Reservoir	1M K/Na tartrate, 0.1M Tris/HCl 7.5, 0.2M Li_2SO_4	25% PEG 3350, 0.1M BisTris/HCl pH 6.5, 0.2M NaCl	1.2M K/Na tartrate, 0.1M MOPS/NaOH pH 8.0, 0.2M Li_2SO_4	1.2M KNaTartrat, 0.1M Tris/HCl pH 7.0, 0.2M Li_2SO_4	3.5M Na formate, 0.1M CHES/NaOH pH 9.5	0.1M NaOAc pH 4.5, 2.5M NaCl, 0.2M Li_2SO_4
Drop volume / protein content	0.5ul / 50%	0.4ul / 50%	0.5ul / 60%	0.6ul / 50%	0.4ul / 70%	0.4ul / 70%
Remarks	Seeding with SAM cocrystals	-	Seeding with SAH-cocrystals	Seeding	-	- / ureido-benzamidine

[1)] Anion bound at carboxylate site of SAM/SAH.

[2)] In diluted protein, prior to concentration by ultrafiltration.

[3)] 1 mM in concentrated protein prior to setup.

[4)] In concentrated protein prior to setup.

Table S2 Data collection and refinement statistics

COMT Crystal<br># / PDB-ID	Human apo<br>(1) / 4PYI	Human apo single swap<br>(2) / 4PYJ	Human apo double swap<br>(3) / 4PYK	Mouse apo<br>(4) / 4P7F	Rat apo Phosphate<br>(5) / 4P7G	Rat apo Sulphate<br>(6) / 4P7J	Rat Sinefungin<br>(7) / 4P7K	rCOMT Sinefungin Tolcapone<br>(8) / 4PYL	hrCOMT apo-Sulphate<br>(9) / 4PYM	hrCOMT SAH<br>(10) / 4PYN	hrCOMT SAH, semi-holo<br>(11) / 4PYO	hrCOMT Ureido-benzamidine<br>(12) / 4PYQ
Detector	MARCCD-225	PILATUS 6M	PILATUS 6M	PILATUS 6M	MARCCD-225	MARCCD-225	MARCCD-225	MARCCD-225	PILATUS 6M	MARCCD-225	PILATUS 6M	PILATUS 6M
Range / Δϕ (°)	125 / 1	180 / 0.5	180 / 1	180 / 0.5	120 / 0.5	180 / 1	180 / 1	133 / 0.3	120 / 0.5	120 / 1	180 / 0.5	180 / 0.5
Mosaicity (°) [0]	0.24	0.10	0.15	0.15	0.41	0.28	0.15	0.82	0.21	0.15	0.13	0.27
Resolution range (Å) [1]	28.8 - 1.35 (1.40 - 1.35)	40.4 - 1.9 (1.97 - 1.9)	40.2 - 2.22 (2.30 - 2.22)	40.8 - 1.37 (1.42 - 1.37)	48.7 - 2.58 (2.67 - 2.58)	45.1 - 1.45 (1.50 - 1.45)	31.7 - 1.22 (1.26 - 1.22)	41.8 - 2.2 (2.28 - 2.2)	30.7 - 1.19 (1.23 - 1.19)	31.7 - 1.2 (1.24 - 1.2)	45.9 - 2.1 (2.17 - 2.1)	36.4 - 1.39 (1.44 - 1.39)
100% crit. (Å) [2]	1.38	1.91	2.22	1.37	2.63	1.45	1.36	2.22	1.21	1.20	2.1	1.42
Space group	P 1	C 2 2 21	C 2 2 21	R 3 2 :H	P 32 2 1	P 31 2 1	P 21 21 21	P 32 2 1	P 21 21 21	P 21 21 21	P 21 21 21	R 3 :H
Cell (Å / °)	31.6, 42.6, 43.7 / 115.0, 95.4, 109.0	54.9, 67.2, 128.8	54.8, 66.8, 128.0	123.8, 88.4	71.6, 393.4	56.3, 119.1	33.2, 61.2, 104.8	49.8, 167.5	33.3, 61.6, 104.2	33.3, 61.2, 104.8	58.4, 60.38, 148.4	125.4, 77.3
Total reflections	101421 (8950)	125270 (12700)	78926 (7716)	544575 (51225)	179257 (8420)	390523 (37495)	302928 (1696)	69025 (3486)	288810 (27852)	305666 (23211)	205887 (20888)	464034 (46477)
Unique reflections	38358 (3366)	18911 (1849)	11951 (1172)	54274 (5362)	36241 (3204)	39464 (3864)	48898 (871)	12916 (1257)	68613 (6699)	67597 (6448)	31280 (3068)	91065 (9131)
Multiplicity	2.6 (2.7)	6.6 (6.9)	6.6 (6.6)	10.0 (9.6)	4.9 (2.6)	9.9 (9.7)	6.2 (1.9)	5.3 (2.8)	4.2 (4.2)	4.5 (3.6)	6.6 (6.8)	5.1 (5.1)
Completeness	92.83	98.60	99.83	99.65	94.84	99.80	75.74	99.65	98.72	99.46	99.58	99.67

(%)	(81.84)	(96.40)	(99.83)	(99.11)	(77.52)	(100.00)	(13.75)	(97.90)	(97.48)	(96.43)	(99.64)	(98.96)
Mean I/σ(I)	6.75 (1.61)	10.76 (1.27)	11.55 (1.53)	14.81 (0.86)	16.13 (1.54)	10.93 (1.87)	28.73 (2.42)	10.12 (1.77)	11.22 (1.08)	10.28 (1.50)	10.24 (1.40)	9.83 (1.49)
Wilson B (Å^2)	10.8	36.4	42.5	18.7	58.2	14.6	9.6	34.8	13.7	8.9	37.5	19.0
R-merge	0.118	0.074	0.104	0.069	0.235	0.117	0.035	0.089	0.059	0.085	0.113	0.075
R-meas	0.147	0.081	0.113	0.073	0.256	0.124	0.038	0.098	0.067	0.096	0.123	0.084
$CC_{1/2}$ [3]	0.989 (0.287)	0.998 (0.737)	0.998 (0.672)	0.999 (0.261)	0.968 (0.676)	0.998 (0.64)	1 (0.769)	0.997 (0.789)	0.999 (0.406)	0.998 (0.496)	0.998 (0.708)	0.997 (0.506)
CC*	0.997 (0.668)	1 (0.921)	1 (0.896)	1 (0.644)	0.992 (0.898)	0.999 (0.883)	1 (0.932)	0.999 (0.939)	1 (0.76)	0.999 (0.814)	1 (0.911)	0.999 (0.82)
R-work [3]	0.1621 (0.3100)	0.1977 (0.3607)	0.1725 (0.2845)	0.1253 (0.3242)	0.2214 (0.3207)	0.1690 (0.2603)	0.1124 (0.2026)	0.1883 (0.2980)	0.1365 (0.2891)	0.1269 (0.2787)	0.1888 (0.3254)	0.1320 (0.3436)
R-free [3]	0.2131 (0.3494)	0.2629 (0.4066)	0.2435 (0.3037)	0.1678 (0.3586)	0.2672 (0.3706)	0.1993 (0.3256)	0.1480 (0.2652)	0.2424 (0.3569)	0.1701 (0.2956)	0.1539 (0.3335)	0.2331 (0.3934)	0.1612 (0.3592)
# molecules / a.u.	1	1	1	1	4	1	1	1	1	1	2	2
# non-H atoms	1804	1693	1723	1980	6843	1972	2176	1765	2086	2136	3524	3667
protein	1666	1619	1670	1759	6725	1750	1836	1677	1762	1809	3414	3383
ligands	1	3	3	31	22	5	17	48	6	4	3	73
water	137	71	50	187	96	217	317	40	313	317	107	211
# residues	207	208	214	213	855	217	219	213	217	219	434	426
RMS (bonds)	0.009	0.006	0.008	0.010	0.014	0.005	0.009	0.008	0.007	0.010	0.007	0.014
RMS (angles)	1.23	0.98	1.07	1.31	1.77	1.13	1.31	1.14	1.21	1.30	1.06	1.30
Ramachandran favoured (%)	98	98	98	96	93	97	97	96	96	97	96	95

Ramachandran outliers (%)	0	0	0	0.44	0.12	0	0	0	0	0	0.24	0.47
Clash score	1.48	3.08	3.29	2.23	3.93	1.13	2.68	5.57	1.70	1.10	3.66	3.63
<B> (Å^2)	18.90	50.90	52.60	26.50	70.30	20.80	13.90	48.30	18.30	12.80	56.10	19.90
protein.	17.90	51.00	52.70	24.80	70.60	19.50	11.40	48.70	16.00	10.70	56.40	19.30
ligands	13.20	47.20	49.90	50.90	70.20	13.80	25.30	41.10	20.10	16.90	86.00	33.90
solvent	30.90	49.50	50.10	37.40	52.50	31.30	27.30	39.50	31.30	24.50	46.40	25.60

[0)] As defined in XDS (Kabsch, 2010).

[1)] Values in parenthesis correspond to the highest resolution shell.

[2)] The 100% criterion was calculated using SFTOOLS (Winn *et al.*, 2011) and represents the resolution in Å of a 100% completed hypothetical data set with the same number of reflections as the measured data.

[3)] R-factors and correlation coefficients were calculated using PHENIX (Zwart *et al.*, 2008). R-values and $CC_{1/2}$ are defined in (Diederichs & Karplus, 1997) and (Karplus & Diederichs, 2012), respectively.

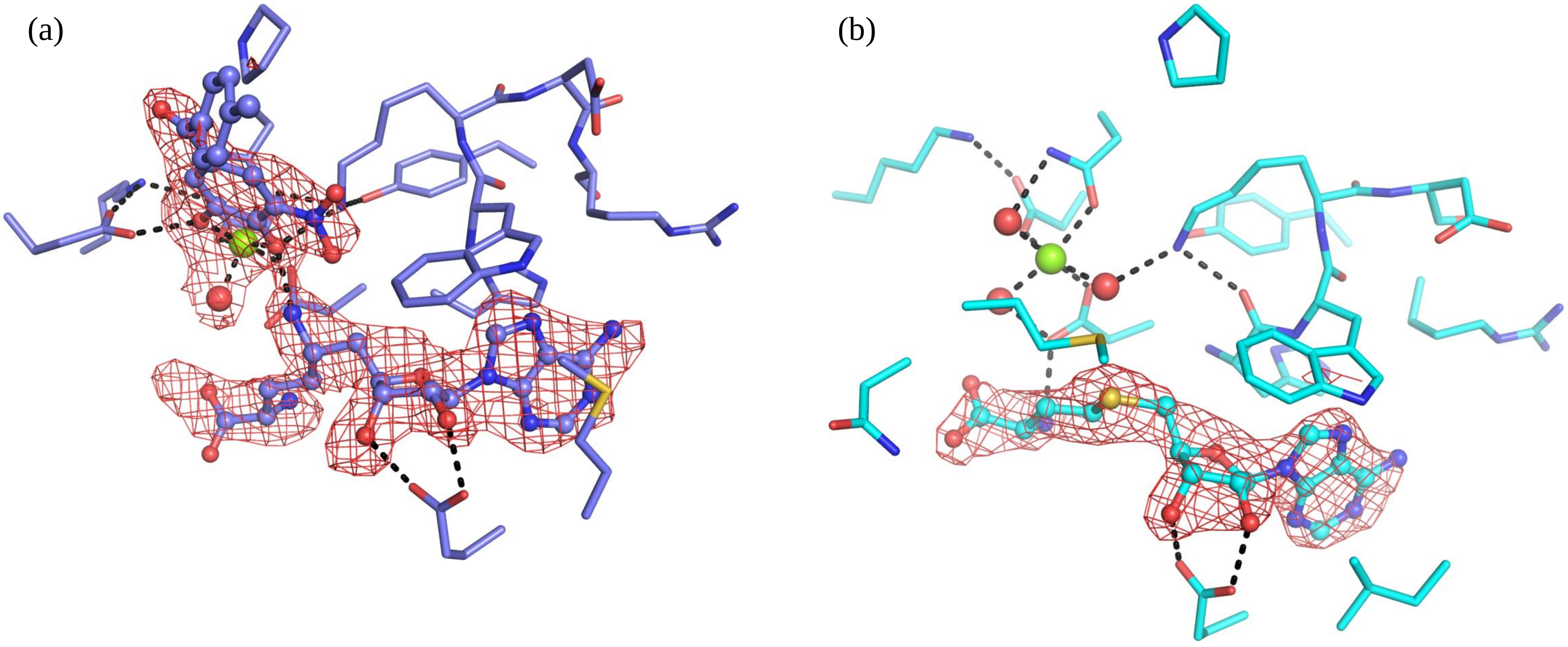

Figure S1 Omit maps of selected ligands, contoured at 3 rmsd and coloured red. (a) Structure **(8)** with sinefungin and tolcapone. (b) Structure **(11b)** with Mg^{2+} and SAH bound. The orientation is the same as in Fig. 6a and b.

```
hCOMT    52 GDTKEQRILNHVLQHAEPGNAQSVLEAIDTYCEQKEWAMNVGDKKGKIVDAVIQEHQPSVLLELGAYCGYSAVRMARLLS
            |...|:|..::||.||.||:...:|..:|.:..:.|:..::|..||:|:..:::|..|:.:||||.|||||.:.:||.|.
hCOMT2   75 GLRIEERAFSYVLTHALPGDPGHILTTLDHWSSRCEYLSHMGPVKGQILMRLVEEKAPACVLELGTYCGYSTLLIARALP

hCOMT   132 PGARLITIEINPDCAAITQRMVDFAGVKD-KVTLVVGASQDIIPQLKKKYDVDTLDMVFLDHWKDRYLPDTLLLEECGLL
            ||.||:|:|.:|..||:.::::..||..: .|.|:||:|:|:||.|:.:|.:...|:|.|.|....||.|..|||...||
hCOMT2  155 PGGRLLTVERDPRTAAVAEKLIRLAGFDEHMVELIVGSSEDVIPCLRTQYQLSRADLVLLAHRPRCYLRDLQLLEAHALL

hCOMT   211 RKGTVLLADNVICPGAPDFLAHVRGSSCFEC-THYQSFLEYREVVDGLEKAIYKGPG 272      38.2 % id.
            ..|..:|||:|:.||||.||.:.:....:.| .|:....::..:.||:.:..|.|||          60.8 % sim.
hCOMT2  225 PAGATVLADHVLFPGAPRFLQYAKSCGRYRCRLHHTGLPDFPAIKDGIAQLTYAGPG 291
```

Figure S2 Sequence alignment of the catalytic domains of human COMT and COMT2. The N-terminal trans-membrane sequences were ignored for the alignment. Sequence numbering for COMT is shifted by fourteen with respect to COMT2. Percent identity and similarity between the sequences are indicated at the end of the alignment. Residues in the adenine and Mg^{2+} binding sites are highlighted with green and cyan backgrounds, respectively. The catalytic lysine residue in COMT and its replacement as proline in COMT2 have a yellow background. A magenta background highlights the residue that binds to the ribose hydroxyl groups (Glu242 in COMT, Asp274 in COMT2).
